# End-to-End Interrupted Sampling Repeater Jamming Countermeasure Network Under Low Signal-to-Noise Ratio

**DOI:** 10.3390/s25133925

**Published:** 2025-06-24

**Authors:** Gane Dai, Xiaoxuan Yang, Sha Huan, Ziyang Chen, Cong Peng, Yuanqin Xu

**Affiliations:** 1 School of Electronic Information Engineering, Foshan University, Foshan 528225, China; daigane@fosu.edu.cn; 2 School of Electronics and Communication Engineering, Guangzhou University, Guangzhou 510006, China; 2112230053@e.gzhu.edu.cn (X.Y.); 2112430060@e.gzhu.edu.cn (Z.C.); 2112430100@e.gzhu.edu.cn (C.P.); 2112430068@e.gzhu.edu.cn (Y.X.)

**Keywords:** end-to-end, complex-valued network, ISRJ suppression, echo reconstruction

## Abstract

Interrupted sampling repeater jamming (ISRJ) is characterized by its coherent processing gains and flexible modulation techniques. ISRJ generates spurious targets along the range, which presents significant challenges to the radar systems. However, existing ISRJ countermeasure methods struggle to eliminate ISRJ signals without compromising the integrity of the real target signal, especially under low-signal-to-noise-ratio (SNR) conditions, resulting in a deteriorated sidelobe and diminished detection performance. We propose a complex-valued encoder–decoder network (CVEDNet) to address these challenges based on signal decomposition. This network offers an end-to-end ISRJ suppression approach, working on complex-valued time-domain signals without the need for additional preprocessing. The encoding and decoding structure suppresses noise components and obtains more compact echo feature representations through layer-by-layer compression and reconstruction. A stacked dual-branch structure and multi-scale dilated convolutions are adopted to further separate the echo signal and ISRJ based on high-dimensional features. A multi-domain combined loss function integrates the waveform and range-pulse-compression information to ensure the amplitude and phase integrity of the reconstructed echo waveform during the training process. The effectiveness of the proposed method was validated in terms of its jamming suppression capability, echo fidelity, and detection performance indicators under low-SNR conditions compared to conventional methods.

## 1. Introduction

Radar technology uses electromagnetic waves to detect targets. It is important for the military, aerospace, traffic monitoring, meteorological observation, and remote sensing [1,2,3,4]. The rapid development of radar technology has introduced more possibilities and more challenges for the civil and military fields. In the realm of military applications, the intricate electromagnetic environment within electronic countermeasures significantly impacts radar detection performance [5,6].

Interrupted sampling repeater jamming (ISRJ) is executed using a store-and-forward methodology [7,8]. It can create multiple false targets, distributed along the range axis, causing tracking errors or even failure. By operating as a coherent jamming technique, ISRJ capitalizes on the processing gain obtained from coherent pulse compression while utilizing reduced transmission power levels. Typically, an ISRJ jammer operates within the duration of a single radar pulse, rendering countermeasures based on inter-pulse diversity ineffective [9,10]. Furthermore, by flexibly adjusting the jamming parameters, diverse jamming effects can be achieved, which will alter the distribution of false targets.

Researchers have proposed methods to reduce the coherence of the ISRJ signal by improving the waveform diversity or modifying the filter kernel function. For instance, the authors of [11] proposed a dual-parameter agile waveform within the pulse to increase the distinction between the radar signal and the ISRJ signal, subsequently employing a parallel suppression technique rooted in the fractional Fourier transform. Similarly, the authors of [12] designed a mismatch filter specifically for the jamming signal to minimize the peak level of the false target. An improved alternating projection algorithm was used for optimization. However, for ISRJ, whose interference parameters can be changed flexibly, this approach necessitates the recalibration of the mismatch filter based on the incoming signal, leading to a reduction in the real-time effectiveness of the countermeasure. Moreover, the mismatched filter kernel reduces the coherence between the filter kernel and the real target signal, leading to energy loss on the range profile.

In addition to the waveform and match filter design, the reconstruction and cancellation approach has emerged as a primary focus of research in this domain. Over time, various parameter estimation methods have been developed to reconstruct ISRJ signals. In [13], Zhou Chao employed the time–frequency analysis of the pulse-compression outcomes to determine the number of slices and forwarding intervals. Subsequently, the slice width was estimated using deconvolution. The jamming signals were reconstructed in the range domain and canceled. However, the mathematical model of the jamming slice introduces errors, due to the approximation of the stationary phase principle in this method, inevitably leading to residual jamming signals. In [14], a method combining time-domain deconvolution with box edge estimation differences was proposed to enhance the accuracy of jamming edge positioning estimation. To minimize the jamming residual, the jamming signal was reconstructed in the time domain. In [15], the delay and distribution of the jamming slices were determined by employing a minimum residual energy criterion, achieved through a search within the truncated matched filter matrix. Additionally, in [16], a one-dimensional semi-parametric technique was utilized to separate target echoes from the received jammed signal. An improved k-means clustering algorithm was then applied to distinguish the jamming signals from the target echoes, enabling the effective reconstruction of the jamming signal. While the aforementioned approaches are well designed and introduce several innovative concepts to the realm of signal processing, the ultimate effectiveness of jamming countermeasures is greatly reliant on the precision of parameter estimation. Ensuring the anti-interference performance proves challenging in low-signal-to-noise-ratio (SNR) conditions.

With the powerful feature extraction capabilities of deep learning, some jamming suppression schemes based on deep learning have emerged in recent years. The authors of [17,18] employed neural networks, such as autoencoders, BiGRU, and GRU, to create masks for jamming-free signals. These jamming-free signal fragments are retained, and compressed sensing is used to reconstruct the complete target echo. Similarly, the authors of [19,20] applied YOLO to identify false targets and real targets in the time–frequency image and extracted the time-range and frequency-domain information of both. In [21], UT-net was utilized to preliminarily suppress jamming in the time–frequency spectrum, followed by utilizing ResCU-net to repair the signal loss caused by the time–frequency transformation. Compared with traditional methods, deep learning exhibits stronger intelligent fitting capabilities and achieves higher accuracy in scenarios with strong jamming and low-SNR conditions. However, the aforementioned methods require at least two stages for jamming suppression: parameter estimation followed by jamming cancellation. Furthermore, there is a need for further exploration of the potential of deep learning networks.

In recent years, there has been an increase in jamming suppression schemes founded on deep learning, leveraging the robust feature extraction capabilities inherent in this technology. The authors of [22] replaced the matched filtering process with a convolutional neural network (CNN) to predict the range information of targets using complex received signals. This approach modeled the jamming suppression problem solely as a classification task; so, the generated sequence could only indicate whether a real target exists in each distance unit, making it challenging to use the magnitude and phase information of the frame series for subsequent velocity processing.

It is essential to maintain the amplitude and phase of the target echoes while performing ISRJ suppression under different SNR conditions. This paper proposes an end-to-end time-domain complex-valued network for ISRJ suppression called the complex-valued encoder–decoder network (CVEDNet). Without any preprocessing, this end-to-end network inputs the jammed signals and outputs clean echo signals, with both the input and output in complex form in the time domain. The architecture of the network follows an encoder–decoder framework. In the feature selection layer, a stacked dual-branch structure with dilated convolutions is designed to separate ISRJ with the echo based on compact signal features. Meanwhile, a multi-domain combined loss function is developed to reconstruct the target signal while maintaining high fidelity during ISRJ suppression.

The contributions of this paper can be outlined as follows:

- The CVEDNet proposed in this study employs a complex-valued encoding and decoding framework, treating the noise component within the received signal as redundant information and successfully eliminating it. This approach proves beneficial for the subsequent signal separation of echo and ISRJ within high-dimensional features. The input and output of this end-to-end network are both complex-valued signals in the time domain, without the need for signal preprocessing, which simplifies the processing steps and is easy to embed into the existing signal processing framework. The complex-valued network can fully utilize the amplitude and phase characteristics to separate ISRJ and reconstruct accurate echo signals based on noise suppression.
- A stacked dual-branch dilated convolution structure is developed, ensuring the model’s superior ability to decompose the ISRJ and echo based on high-dimensional features. Coherent jamming slices make it difficult to select target information from the received signal. Different dilated ratios are applied to extract features of echo and ISRJ from multiple scales. The stacked dual-branch structure realizes the separation and suppression of ISRJ step by step according to these multiple-scale features.
- A multi-domain combined loss function is proposed considering the difference in appearance of the ISRJ signals in the time-domain and pulse-compression results. The network training process combines waveform similarity and range-pulse-compression similarity to minimize the difference between the reconstructed target signal and clean echo. This improves the integrity of the reconstructed target signal output by the network.

This paper is structured as follows. Section 2 analyzes the jamming mechanism of ISRJ and establishes the mathematical model of the signals jammed by ISRJ. Section 3 describes the proposed CVEDNet. Section 4 provides some experimental verification of the proposed network. Finally, Section 5 offers conclusions.

## 2. Mathematical Model of the Signals Jammed by ISRJ

The radar waveform employed in this paper is the linear frequency modulation (LFM) signal, which is widely adopted in radar systems due to its superior time–bandwidth product. The high time–bandwidth product enhances the maximum detection range of radar targets while significantly improving the range resolution, making the LFM signal a preferred choice for radar detection applications.

The mathematical expression of the echo signal after down-conversion is as follows:

(1) $$s\left(t\right)=A_{t}\mathrm{rect}\left(\frac{t-\tau_{t}}{T_{c}}\right)\exp\left\{-j2\pi f_{c}\tau_{t}\right\}\exp\left\{j\pi \gamma {\left(t-\tau_{t}\right)}^{2}\right\},$$

where $A_{t}$ characterizes the signal amplitude, *t* refers to the temporal dimension of the signal, $\tau_{t}$ indicates the time delay of the target echo, $T_{c}$ specifies the pulse duration, $f_{c}$ corresponds to the carrier frequency, and $\gamma =\frac{B_{c}}{T_{c}}$ defines the chirp rate. The parameter $B_{c}$ specifies the bandwidth.

Given that the target distance to be measured is $R_{t}$, the time delay $\tau_{t}$ is expressed as

(2) $$\tau_{t}=\frac{2R_{t}}{c},$$

where c is the speed of light.

The ISRJ jamming mechanism allows it to quickly generate multiple false targets along the range direction through high coherence. The jammer captures the radar signal and retransmits some signal segments according to a predefined strategy.

**Figure 1:**
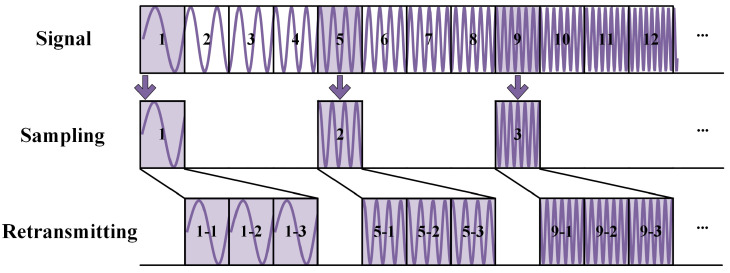
Diagram of the ISRJ mechanism.

As shown in Figure 1, the mathematical model of the ISRJ signal can be written as

(3) $$\begin{array}{cc} J_{ISRJ}\left(t\right)= & A_{j}\sum_{n=0}^{N-1}\sum_{m=0}^{M-1}\mathrm{rect}\left(\frac{t-nT_{u}-mT_{s}-\tau_{j}}{T_{s}}\right)\\ \; & \exp\left\{-j2\pi f_{c}\left(mT_{s}+\tau_{j}\right)\right\}\exp\left\{j\pi \gamma {\left(t-mT_{s}-\tau_{j}\right)}^{2}\right\}, \end{array}$$

where $A_{j}$ represents the amplitude of ISRJ, *N* is the number of sampling instances of the jamming, and *M* is the number of forwarding times. $T_{u}$ is the time interval for each interception, $T_{s}$ is the slice width, and $\tau_{j}$ is the delay from the jammer to the radar receiver. For the sake of convenience, the processing delay incurred between the jammer intercepting the radar signal and transmitting the jamming signal is disregarded.

Therefore, when there are target signals and jamming signals within the radar observation range, the jammed signal obtained by the radar receiver can be expressed as

(4) $$x\left(t\right)=s\left(t\right)+J_{ISRJ}\left(t\right)+n\left(t\right).$$

In the above equation, n(t) represents the noise. The received signal, including ISRJ, is a superposition of multiple components in the time domain.

## 3. ISRJ Suppression Network

Considering that deep networks can be used for signal separation, this paper proposes the CVEDNet for ISRJ suppression, which eliminates interference and noise components based on high-dimensional features and reconstructs the waveform of the target echoes in the time domain.

### 3.1. ISRJ Suppression Process

**Figure 2:**
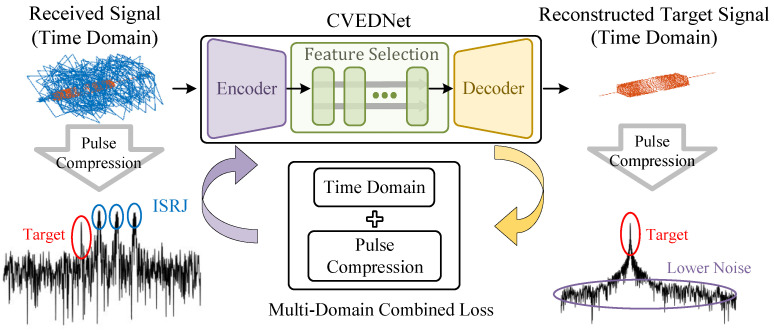
Schematic diagram of the proposed ISRJ suppression network.

The diagram of the ISRJ suppression process is shown in Figure 2. The down-converted signal within the radar reference range is collected in the time domain. This signal works as the input of the CVEDNet in complex-valued form, and the network outputs the reconstructed complex-valued target signal also in the time domain, achieving the goal of filtering out ISRJ and noise from the received signal.

The CVEDNet was designed with three parts to complete the task of ISRJ suppression.

1. Encoder module. The encoder module can better characterize the internal structure and characteristics of the received signal through feature transformation, thus improving its representation. It ignores noise as unimportant information and focuses on the amplitude and phase characteristics of the target signals and ISRJ. This operation is beneficial for the subsequent models to extract compact features.
2. Feature selection module. This module uses a stacked dual-branch structure and multi-scale dilated convolution to select the target echo features from the received signals, suppressing ISRJ.
3. Decoder module. It receives the amplitude and phase characteristics of the target signals extracted by the feature selection module and uses deconvolution to reconstruct the complex-valued echo in the time domain.

During the training process of the network, a multi-domain combined loss function, integrating both time-domain and pulse-compression-domain information, is employed to supervise the model’s fitting accuracy from the perspectives of the time-domain error and the linear frequency modulation correlation.

### 3.2. CVEDNet

#### 3.2.1. Overall Network Structure

As the network operates on discrete data, the analog signal in Equation (4) must be converted into a complex-valued vector.

We use $s\in C^{L\times 1}$ to represent the target signal vector in the time domain, where

(5) $$s={\left[\begin{array}{c} s(1),\ldots ,s(L) \end{array}\right]}^{T}.$$

s(l)∈C denotes the *l*-th complex-valued sample of the target signal for l=1,…,L. *L* represents the vector length.

Similarly, $x\in C^{L\times 1}$ represents the received signal vector including the ISRJ and noise, where

(6) $$x={\left[\begin{array}{c} x(1),\ldots ,x(L) \end{array}\right]}^{T}.$$

x(l)∈C denotes the *l*-th complex-valued sample of the received signal for l=1,…,L.

In this study, ISRJ suppression is defined as a signal reconstruction task. The clean echo waveform is restored from the received signal x so that its amplitude and phase information are as consistent as possible with s. The reconstructed complex-valued vector in the time domain is represented by $\tilde{s}\in C^{L\times 1}$.

**Figure 3:**
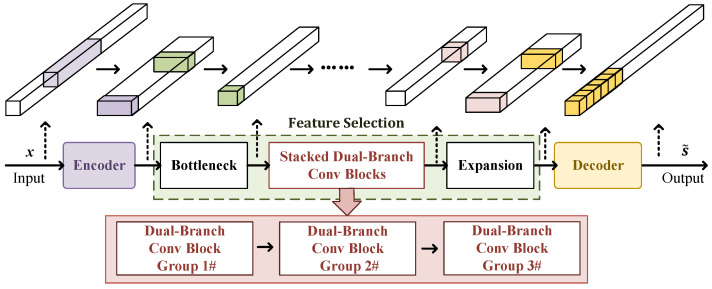
Diagram of the CVEDNet.

The architecture of the proposed CVEDNet is depicted in Figure 3. Within the CVEDNet, the encoder module extracts a set of embedding features from the input signal and suppresses noise.

The extracted embedding features obtained from the encoder module are processed by the feature selection layer in the subsequent stages. The feature selection layer is composed of the BottleNeck block, stacked dual-branch convolution blocks (DBCBs), and an expansion block. The BottleNeck and expansion blocks are used in pairs to achieve channel conversion through point convolution operations. The BottleNeck block is used to reduce the number of channels output by the encoder block, while the expansion block is used to increase the number of channels of the feature selection layer output. They effectively connect the feature selection layer and the encoding and decoding structures. The stacked DBCBs effectively separate the ISRJ components while preserving the features associated with the clean target signal from the high-dimensional features. A certain number of two-branch convolution kernels constitute a group from different scales, and three groups are repeated to form the stacked two-branch convolution structure.

Finally, the decoder converts the high-dimensional characteristics of the target signal without ISRJ into a complex-valued time-domain signal.

The CVEDNet is a complex-valued network. Therefore, both convolution and non-convolution operations need to support complex-valued data. Complex-valued convolution (CVConv) involves operations on the real and imaginary parts of the input data and the convolution kernel. The operation process of Y=W∗X is shown as follows:

(7) $$\left[\begin{array}{c} Y_{r}\\ Y_{i} \end{array}\right]=\left[\begin{array}{cc} W_{r} & -W_{i}\\ W_{i} & W_{r} \end{array}\right]*\left[\begin{array}{c} X_{r}\\ X_{i} \end{array}\right].$$

The subscripts *r* and *i* represent the real and imaginary parts, respectively.

Non-convolution operations in the network model use parallel calculations of the real part and the imaginary part. The mathematical model of Y=f(X) is as follows:

(8) $$\left[\begin{array}{c} Y_{r}\\ Y_{i} \end{array}\right]=f\left(\left[\begin{array}{c} X_{r}\\ X_{i} \end{array}\right]\right)=\left[\begin{array}{c} f\left(X_{r}\right)\\ f\left(X_{i}\right) \end{array}\right].$$

Among them, $f\left(\cdot \right)$ represents the non-convolution operation in the network structure, such as the concat operation, activation function, pooling function, etc.

All the convolution and non-convolution operations mentioned below are complex-valued operations.

#### 3.2.2. The Encoding Module

The encoder is a critical component within the CVEDNet structure. It is responsible for transforming the input waveform into high-dimensional features through convolution operations at two different scales. This transformation increases the spatial dimension of the extracted embedding features.

**Figure 4:**
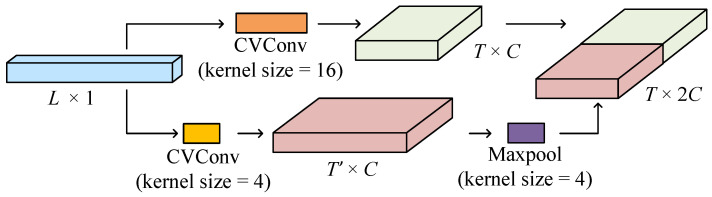
Illustration of the encoder block.

The structure of the encoder is illustrated in Figure 4. The encoder utilizes two scales of complex-valued convolution kernels to extract features from an input vector x. The CVConv operation at scale *k* is defined as the operation that applies the convolution kernel $w_{k}$ to input signal x. This can be expressed as

(9) $$Y_{k}=x*w_{k},\;\mathrm{for}\;k=4,16.$$

In the above equation, $w_{k}\in C^{k\times 1}$ represents the complex-valued convolution kernel of size k×1 as a vector. The features are extracted from the input at two different scales, k=4 and k=16, using parallel branches.

To ensure compatibility in spatial dimensions between the two branches, a maxpooling operation with a stride of 2 is applied to the branch with a kernel size of 4×1. This operation can be mathematically expressed as

(10) $$Y_{4}^{\mathrm{down}}=\mathrm{maxpool}\left(Y_{4}\right)\;\mathrm{with}\;\mathrm{stride}=2,$$

where $Y_{4}^{\mathrm{down}}$ represents the output of the maxpool operation. Here, the stride parameter indicates the step size of the pooling operation, which determines the distance by which the pooling window shifts during the process. After maxpooling, the spatial dimensions of both feature maps are aligned, allowing for concatenation along the channel dimension.

The features from both branches are concatenated along the channel dimension after the data dimensions of the two branches are aligned. This operation can be denoted as

(11) $$Y_{\mathrm{concat}}=\mathrm{concat}\left(Y_{4}^{\mathrm{down}},Y_{16}\right).$$

#### 3.2.3. The Feature Selection Module

The feature selection module is composed of the bottleneck, stacked DBCBs, and the expansion module.

Implemented using pointwise convolution layers, the bottleneck and expansion modules enable the model to linearly combine channel features at each spatial position, facilitating the extraction of richer and more diverse features. These modules play a critical role in compressing and expanding the channel features within the CVEDNet architecture, enhancing the network’s representational capacity and computational efficiency.

Mathematically, the operation of the bottleneck and expansion modules can be expressed as

(12) $$H^{\prime }=HW+b,$$

where H represents the input feature tensor, W is the weight matrix of the pointwise convolution, and b is the bias term. The output $H^{\prime }$ contains the transformed feature representations.

**Figure 5:**
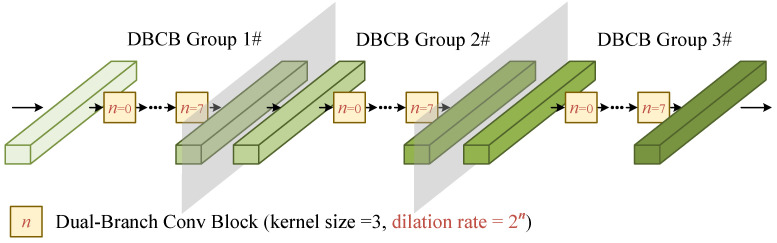
Schematic illustration of stacked DBCBs.

**Figure 6:**
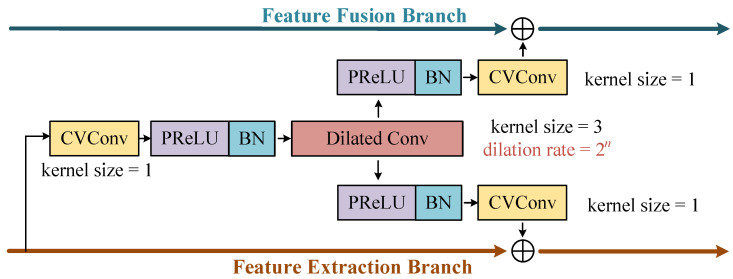
Schematic illustration of the DBCB.

The stacked DBCBs, as shown in Figure 5, are constructed by three identical DBCB groups. Each group consists of eight DBCBs with dilation rates ranging from $2^{0}$ to $2^{7}$. The network structure of the DBCB is provided in detail in Figure 6.

The DBCBs capture complex features across various scales for the accurate target signal feature selection. DBCB has two signal paths, corresponding to a pair of inputs and outputs. As shown in Figure 6, the upper branch is called the feature fusion branch, and the lower branch is called the feature extraction branch. The output of the feature extraction branch of the previous DCBC will be convolved according to the dilation ratio of the current DBCB to generate two intermediate features. One of the intermediate feature outputs is added to the input of this branch through the residual structure to generate the output of the feature extraction branch of the current DBCB. The other intermediate feature is added to the input of the feature fusion branch to generate the output of the feature fusion branch of this DBCB.

**Figure 7:**
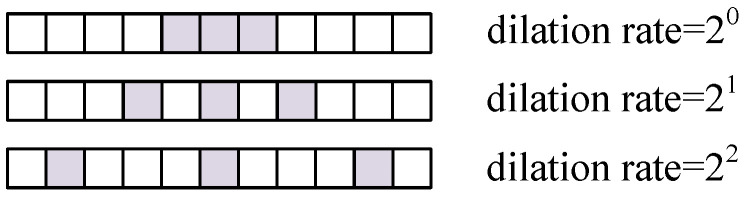
Diagram of the dilated convolution kernel.

The convolution processing of DBCB includes complex-valued convolutions, parametric rectified linear unit (PReLU) modules, batch normalization modules (BN), and the dilated convolutions of different ratios. The dilated convolution can adjust the receptive field by setting a different dilation ratio, achieving the extraction of features on different scales. Unlike traditional convolution operations that require enlarging the receptive field by increasing the size of the convolution kernel, dilated convolution can expand the receptive field without altering the number of parameters. It can reduce the convolution operation and improve the overall computational efficiency. A convolution kernel with a smaller dilation rate is beneficial for capturing intricate details in the signal, while a kernel with a larger dilation rate is advantageous for extracting broad global features within the signal. The input and output data sizes of the DCBC remain consistent. Figure 7 presents a schematic diagram of the distribution of the dilation convolution kernel when the dilation rate is $2^{0}$, $2^{1}$, and $2^{2}$, and the kernel size is 3.

#### 3.2.4. The Decoder Module

In the decoding process, the deconvolutional operations symmetrically reverse the encoding transformations, restoring the temporal resolution and feature dimensionality of the input signal. This ensures that the final output retains the key structural and semantic information from the original target echo, with minimized information loss. The deconvolution operation can be expressed as

(13) $$\tilde{s}=\mathrm{padding}\left(H^{\prime }\right)*W_{k},\;\mathrm{for}\;k=16,$$

where $W_{k}$ is the deconvolution kernel. The padding operation ensures that the feature map $H^{\prime }$ is expanded appropriately before applying the deconvolution.

### 3.3. Multi-Domain Combined Loss Function

The superior performance of convolutional networks has been extensively validated through their wide application in the field of signal processing. However, in many cases, achieving high training accuracy is difficult, due to the inability of traditional loss functions to provide a smooth optimization trajectory for the model. Loss functions that rely on a single indicator often suffer from being overly one-dimensional and fail to capture the complexity of the training process. Consequently, loss functions that integrate multiple indicators have become indispensable tools for training high-performing models.

A loss function that combines amplitude loss and waveform perception loss enables the model to account for both amplitude and waveform errors during training, thereby providing a more comprehensive evaluation of waveform reconstruction. In recent years, such loss functions, which incorporate error and perceptual components, have become increasingly prevalent in generative models, including the widely recognized stable diffusion [23] and speech generation models [24].

In this study, the mean squared error (MSE) in the time domain and the perceptual loss in the pulse-compression domain are combined to evaluate the waveform similarity and reconstructed signal coherence.

The MSE is defined to quantify the squared difference between the reconstructed signal and the reference signal, as expressed below:

(14) $$L_{MSE}=\frac{1}{L}{∥\tilde{s}-s∥}_{2}^{2}.$$

The pulse-compression process is defined as follows. We assume that the reference signal $s_{\mathrm{ref}}$ is the time-reversed complex conjugate of the radar signal.

The output of the pulse-compression process is derived by conducting an inverse Fourier transform (IFFT) on the product of the Fourier transforms (FFTs) of the target echo s and the reference signal $s_{\mathrm{ref}}$. Mathematically, the amplitude of the pulse-compression result can be written as

(15) $$s_{\mathrm{pc}}=\mathrm{abs}\left(\mathrm{IFFT}\left(\mathrm{FFT}\left(s\right)\cdot \mathrm{FFT}\left(s_{\mathrm{ref}}\right)\right)\right).$$

Here, $\mathrm{abs}\left(\cdot \right)$ represents the absolute value operation.

The network reconstructed time-domain waveform is also converted to the pulse-compression domain and expressed as

(16) $${\tilde{s}}_{\mathrm{pc}}=\mathrm{abs}\left(\mathrm{IFFT}\left(\mathrm{FFT}\left(\tilde{s}\right)\cdot \mathrm{FFT}\left(s_{\mathrm{ref}}\right)\right)\right).$$

The decibel of $s_{\mathrm{pc}}$ is expressed as

(17) $$s_{\mathrm{dB}}=20\log_{10}\left(s_{\mathrm{pc}}\right).$$

Similarly, the decibel of ${\tilde{s}}_{\mathrm{pc}}$ can be defined as ${\tilde{s}}_{\mathrm{dB}}$.

The perceptual loss is formulated based on the output of pulse compression and is defined as

(18) $$L_{\mathrm{perceptual}}=\alpha {∥s_{\mathrm{dB}}-{\tilde{s}}_{\mathrm{dB}}∥}_{1}+\beta \frac{∥s_{\mathrm{pc}}-{\tilde{s}}_{\mathrm{pc}}{∥}_{2}}{∥s_{\mathrm{pc}}{∥}_{2}},$$

where ${∥\cdot ∥}_{1}$ denotes the $L_{1}$-norm and is used to quantify the absolute error, while ${∥\cdot ∥}_{2}$ denotes the $L_{2}$-norm and is employed to measure the relative error. α and β are two balancing parameters.

Finally, the combined loss function is designed as follows:

(19) $$L=L_{MSE}+L_{\mathrm{perceptual}}.$$

## 4. Experiments

### 4.1. Experiment Settings

To assess the efficacy of the proposed network, we used Matlab simulation to establish a dataset of radar echo waveforms with interference under different SNRs. The radar was assumed to be a pulse radar operating at a carrier frequency of 16 GHz. The detection distance was in the range of 0 to 30 km. It was assumed that the jammer and the target were at the same location. To simulate different levels of jamming strength relative to the target signal, the jamming-to-signal ratio (JSR) was defined within the range of 0 to 50 dB. The time width of a single jamming slice was between 0.512 µs and 1.536 µs, and the number of jamming repetitions in each pulse was between 2 and 5. The parameters were randomly selected within this range to generate an echo dataset with jamming to train the network. The noise type added to the signal in the simulation is Gaussian white noise. It is assumed that the frequency response is ideal during the waveform transmission, jammer processing, and radar system processing. The simulation parameters are detailed in Table 1.

Following the parameter range outlined in Table 1 and the ISRJ pattern illustrated in Figure 1, a total of 30,420 data samples have been generated. Each data sample is a single frame of time-domain complex baseband echo data, including ISRJ and noise. A random initial phase is added to the echo in each data sample.

A specific example of the parameters related to the CVEDNet, including the details of each layer, its corresponding output size, and the number of parameters, is provided in Table 2. The network’s code has been uploaded to GitHub and is available at https://github.com/chai-xuan/CVEDNet (accessed on 20 March 2025).

### 4.2. Evaluation Metrics

#### 4.2.1. Time-Domain Evaluation Metrics

The input and output signals of the CVEDNet network are both time-domain signals. The waveform reconstruction performance of the proposed network can be directly evaluated in the time domain. Meanwhile, radar signals are transformed to the range domain by pulse compression for target detection. Therefore, different indicators are used to evaluate the jamming suppression performance of the proposed network from both the time domain and the range domain.

To assess the difference between the reconstructed signal and the real target echo, the scale-invariant signal-to-distortion ratio (SISDR) and scale-invariant signal-to-noise ratio (SISNR) are introduced to measure the waveform similarity.

The definition of the SISDR is as follows:

(20) $$\mathrm{SISDR}=10\log_{10}\frac{{∥r(\tilde{s},s)∥}_{2}^{2}}{{∥\tilde{s}-r\left(\tilde{s},s\right)∥}_{2}^{2}}.$$

In the above equation, $r(\tilde{s},s)$ is calculated as

(21) $$r\left(\tilde{s},s\right)=\frac{\langle \tilde{s},s\rangle s}{{∥s∥}_{2}^{2}},$$

where 〈·〉 represents the inner product.

The calculation of the SISNR is similar to the SISDR, with the addition of the de-averaging of the reconstructed signal. The signal obtained by the following de-averaging operation is defined as ${\tilde{s}}_{\mathrm{da}}$. Substituting ${\tilde{s}}_{\mathrm{da}}$ for $\tilde{s}$ in Equation (21) and calculating the result in Equation (20) gives the value of the SISNR.

(22) $${\tilde{s}}_{\mathrm{da}}=\tilde{s}-\mu \left(\tilde{s}\right),$$

where $\mu (\tilde{s})$ denotes the mean of the reconstructed signal, which is given by $\mu \left(\tilde{s}\right)=\frac{1}{L}\sum_{l=1}^{L}\tilde{s}\left[l\right]$. The definition of the SISNR is as follows:

(23) $$\mathrm{SISNR}=10\log_{10}\frac{{∥r({\tilde{s}}_{\mathrm{da}},s)∥}_{2}^{2}}{{∥{\tilde{s}}_{\mathrm{da}}-r\left({\tilde{s}}_{\mathrm{da}},s\right)∥}_{2}^{2}}.$$

The scale-invariant signal-to-distortion ratio improvement factor (SISDR-IF) is defined to evaluate the quality of the waveform before and after the jamming and noise suppression, and it can be calculated as follows:

(24) $$\mathrm{SISDR-IF}=10\log_{10}\frac{{∥r(\tilde{s},s)∥}_{2}^{2}}{{∥\tilde{s}-r\left(\tilde{s},s\right)∥}_{2}^{2}}-10\log_{10}\frac{{∥r(x,s)∥}_{2}^{2}}{{∥x-r(x,s)∥}_{2}^{2}}.$$

#### 4.2.2. Range Domain Evaluation Metrics

The detection capability of radar is evaluated based on the detection probability ($P_{d}$) and false alarm probability ($P_{fa}$), which serve as key indicators to assess the effectiveness of jamming suppression for target detection. The definitions of $P_{d}$ and $P_{fa}$ are provided as follows:

(25) $$P_{d}=\frac{N_{\mathrm{pt}}}{N_{\mathrm{total}}},$$

where $P_{d}$ denotes the probability of detecting true targets. Here, $N_{\mathrm{pt}}$ represents the number of true targets successfully detected, and $N_{\mathrm{total}}$ refers to the total number of true targets.

(26) $$P_{fa}=\frac{N_{\mathrm{pf}}}{N_{\mathrm{total}}}.$$

In the above equation, $P_{fa}$ quantifies the probability of false target detections. Specifically, $N_{\mathrm{pf}}$ represents the number of false targets detected, while $N_{\mathrm{total}}$ is defined as the total number of detected targets in the experiment.

Meanwhile, the signal-to-jamming ratio improvement factor (JSRIF) is used to evaluate the effectiveness of the proposed method in suppressing jamming. The definition of the JSRIF is as follows:

(27) $$\mathrm{JSRIF}={\mathrm{JSR}}_{\mathrm{in}}-{\mathrm{JSR}}_{\mathrm{out}},$$

where ${\mathrm{JSR}}_{\mathrm{in}}$ represents the JSR before the jamming suppression, and ${\mathrm{JSR}}_{\mathrm{out}}$ represents the JSR in the output of CVEDNet.

### 4.3. Training Results of CVEDNet

The model was trained using an NVIDIA RTX 4090 GPU. The learning rate was set to $1\times 10^{-4}$, and the Adam optimizer was used. A total of 1000 training epochs were conducted. The two balance parameters in the combined loss function were α=0.1 and β=1. During the training process of CVEDNet, the dataset was divided into a training set, validation set, and test set according to 8:1:1. A dropout layer with dropout rates of 0.2 and 0.3 was incorporated into each DBCB module to combat potential overfitting issues effectively.

**Figure 8:**
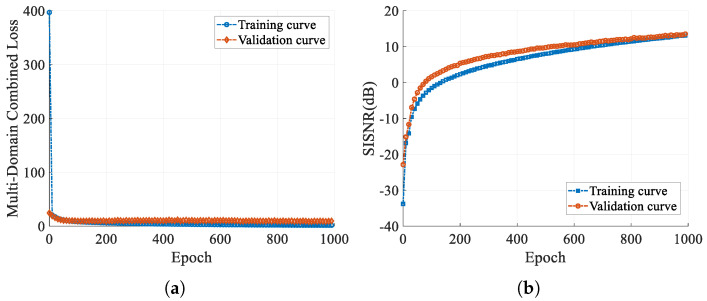
Training curve of the proposed CVEDNet. (**a**) The multi-domain combined loss curve. (**b**) SISNR curve.

The results depicted in Figure 8 demonstrate the good performance of CVEDNet on both the training and validation sets, exhibiting no evidence of overfitting or underfitting. The multi-domain combined loss performance on both sets exhibits a consistent downward trajectory, with no discernible disparities in performance metrics, suggesting the model’s adeptness at effectively capturing training data patterns and generalizing to unseen data. This equilibrium in performance indicates that the model can capture important features while avoiding overfitting the details of the training data, showing good generalization ability and robustness.

### 4.4. Quality Analysis of the ISRJ Suppression and Echo Reconstruction

**Figure 9:**
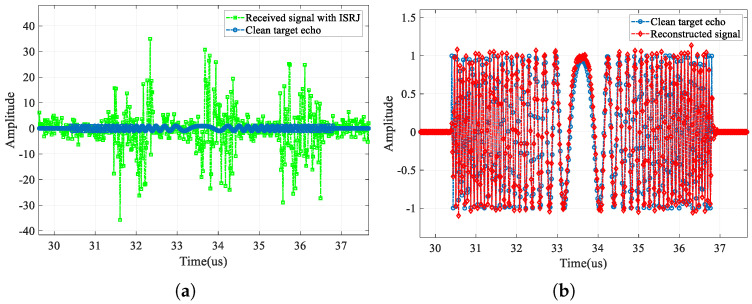
Time-domain signal (real part) comparison when the SNR is −10 dB and the JSR is 8 dB. (**a**) Received signal with ISRJ and clean target echo; (**b**) clean target echo and the reconstructed signal.

**Figure 10:**
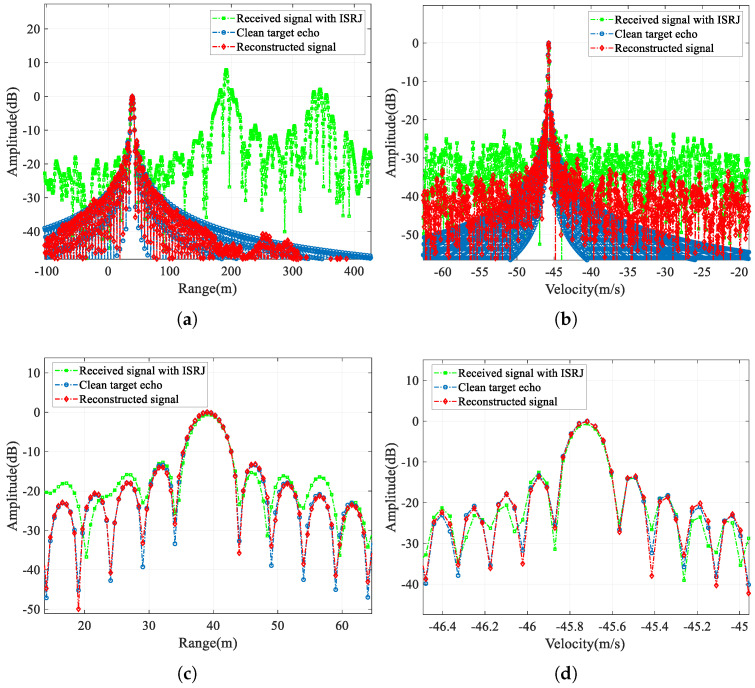
Pulse-compression comparison when the SNR is −10 dB and the JSR is 8 dB. (**a**) Comparison in the range profile, (**b**) comparison in the velocity profile, (**c**) comparison in the enlarged range profile, and (**d**) comparison in the enlarged velocity profile.

First, in this section, we compare the jammed signal, the clean target echo, and the reconstructed signal output by CVEDNet. Their time-domain waveforms (real part) and pulse-compression results are shown in Figure 9 and Figure 10, respectively, when the SNR is −10 dB, and the JSR is 8 dB.

From the time-domain comparison, the ISRJ component with a higher power in the received signal marked in green is effectively eliminated in the red reconstructed signal curve, and the reconstructed waveform matches the clean target echo marked in blue well, even under low-SNR conditions. In the reconstructed signal, not only are the ISRJ components eliminated, but the noise is also suppressed to a considerable extent.

The pulse-compression results compare the jamming suppression performance from the range dimension of the single-frame signal and the velocity dimension of the multi-frame signals. The range pulse compression shows that the peak energy of the false target introduced by ISRJ can be effectively eliminated. The amplitude and position of the target in the reconstructed signal are consistent with those of the true target, which can be clearly observed in the enlarged view of the range mainlobe. The velocity-pulse-compression results illustrate the phase fidelity performance of the CVEDNet based on complex-value operations; so, the pulse-compression results among the multiple reconstructed signals can still be consistent with the true target velocity.

Table 3 quantitatively evaluates the signal quality of the reconstructed echo signal output by the CVEDNet. Some evaluation indicators of the original echo signal and the reconstructed waveform, such as the peak sidelobe ratio (PSLR) and integrated sidelobe ratio (ISLR), are listed in the table. The results show that CVEDNet can achieve fidelity waveform reconstruction while effectively suppressing the ISRJ. The PSLR of range compression only drops by 0.25 dB, indicating the high quality of the reconstructed waveform. The velocity-pulse-compression results are almost consistent with the original target information, showing that the network has a strong ability to maintain the phase information.

### 4.5. Evaluation of the Proposed Loss Function

To validate the effectiveness of the proposed loss function in ISRJ suppression, some commonly used loss functions were used for model training as comparisons. The training process adopted the same model architectures and training step sizes. Table 4 presents the comparison of different loss functions according to some time-domain evaluation metrics.

Table 4 shows the evaluations of three loss functions as comparisons. The results demonstrate that the multi-domain combined loss function achieved superior performance across all four metrics. In the SISDR, the combined multi-domain loss reached a value of 14.13 dB, higher than that of the other loss functions.

In the de-averaged performance comparisons, taking the SISNR-IF as an example, the indicators of the multi-domain combined loss function were improved by 1.4 dB, 0.67 dB, and 0.88 dB, respectively, compared with the MSE, MSE+MAE, and MSE+Smooth L1. These results conclusively demonstrate that the multi-domain combined loss function outperforms alternative loss functions, highlighting its potential in ISRJ suppression.

### 4.6. Robustness to the Jamming-Free Duty Cycle

In the following sections, we present the assessment of the detection and ISRJ suppression performance, attained from around $10^{5}$ Monte Carlo simulations.

CVEDNet receives the complex-valued signal with ISRJ in the time domain, suppresses the jamming parts, and outputs the reconstructed complex-valued echo waveform. We evaluated the impact of the ISRJ duty cycle on the jamming suppression and echo reconstruction performance of CVEDNet. η represents the proportion of jamming-free time within the time domain where the target echo exists, which is defined as follows:

(28) $$\eta =1-\frac{MNT_{s}}{T_{c}}.$$

**Figure 11:**
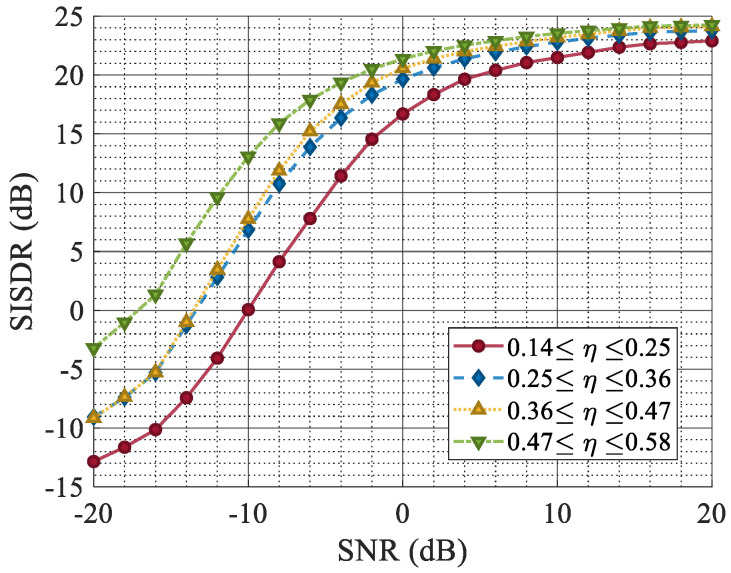
The SISDR versus the SNR under different ISRJ duty cycles.

Figure 11 shows the SISDR, which was used to measure the error between the original target echo and the reconstructed signal under different SNRs. Signals with different jamming ratios were divided into four intervals according to η, among which the purple curve represents the signal with the smallest jamming ratio, and the blue curve represents the signal with the highest jamming ratio.

The simulation results indicate that with a high proportion of ISRJ, the quality of waveform reconstruction decreases, albeit to a small extent. The quality of the waveform reconstruction is essentially unaffected by the proportion of jamming under a high SNR. The overall quality of the waveform reconstruction is directly correlated with the SNR. When the SNR is −10 dB, and the jamming ratio is around 50%, the SISDR is 12.5 dB. These metrics stabilize when the SNR exceeds −10 dB.

**Figure 12:**
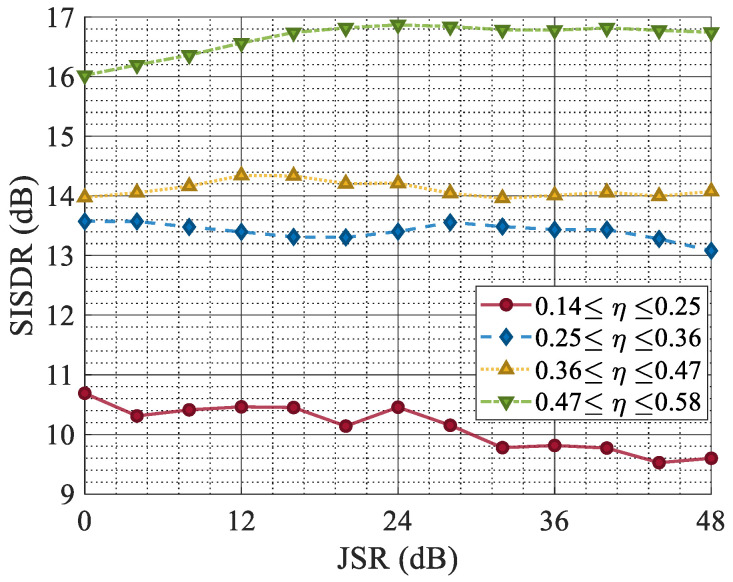
The SISDR versus the JSR under different ISRJ duty cycles.

Figure 12 shows the echo reconstruction performance of the proposed ISRJ countermeasure network under the condition of varying the jamming power. As the JSR increases from 0 dB to 50 dB, the simulation results indicate that the jamming power minimally impacts the quality of the reconstructed waveform. Upon successful and comprehensive extraction of the features of the ISRJ signal, effective jamming separation and suppression can be attained.

**Figure 13:**
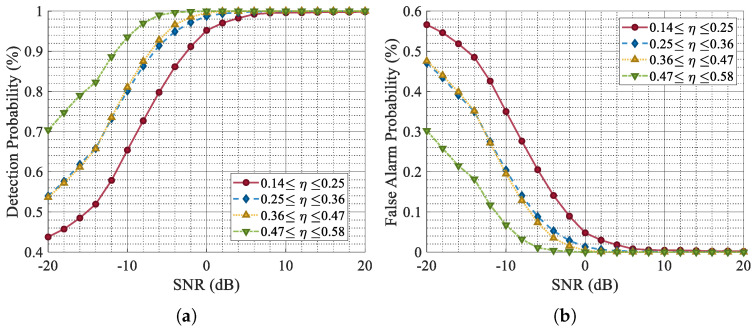
Comparison of $P_{d}$ and $P_{fa}$ with different η. (**a**) $P_{d}$ for signals categorized by η. (**b**) $P_{fa}$ for signals categorized by η.

The simulation results of the target detection performance for different values of η also support a similar conclusion, as shown in Figure 13. At an SNR of −10 dB, signals with a jamming ratio around 50% achieve detection probabilities of over 90% after ISRJ suppression and waveform reconstruction by CVEDNet.

### 4.7. Comparison with Other Methods

To further demonstrate the robustness and superiority of the proposed network, we compared the jamming suppression network presented in this study with MaxTF [26], BCS [27], and MMF [12] across various metrics.

**Figure 14:**
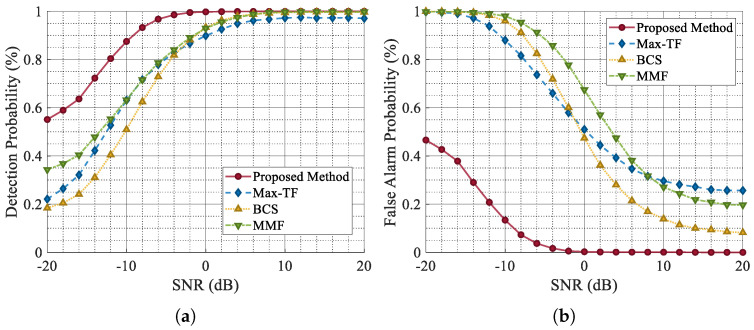
Comparison of $P_{d}$ and $P_{fa}$ for different anti-jamming methods under varying SNR conditions. (**a**) $P_{d}$ for the comparison. (**b**) $P_{fa}$ for the comparison.

From the experimental results shown in Figure 14, it is evident that as the SNR decreases, the $P_{d}$ of each scheme deteriorates to varying degrees, while the $P_{fa}$ correspondingly increases. However, the jamming suppression network proposed herein demonstrates the ability to maintain a high $P_{d}$ and a low $P_{fa}$ under SNR conditions of around −10 dB. Compared with other schemes, the proposed method consistently delivers superior jamming suppression performance in low-SNR scenarios. The improved false alarm performance also demonstrates the capability of this network to effectively mitigate ISRJ and noise.

**Figure 15:**
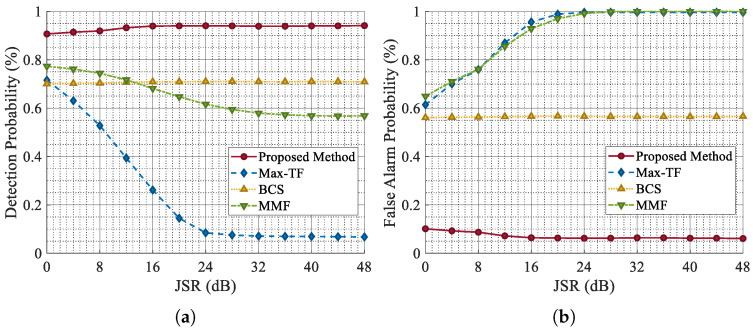
Comparison of $P_{d}$ and $P_{fa}$ for different anti-jamming methods under varying JSR conditions. (**a**) $P_{d}$ for the comparison. (**b**) $P_{fa}$ for the comparison.

As demonstrated in Figure 15, the $P_{d}$ values of MMF and Max-TF gradually decrease with the increase in the JSR, which shows that they are sensitive to the strength of the jamming signal and find it difficult to maintain the original jamming suppression performance as the jamming strength increases. This is because both MMF and Max-TF employ adaptive filters for ISRJ suppression. When the jamming power level is high, the residual jamming energy is also substantial, which can degrade the performance of target detection. In contrast, the $P_{d}$ and $P_{fa}$ values of BCS and the proposed method maintain stable performance across different JSR levels, proving their robustness to varying jamming strengths. Nevertheless, the BCS method is sensitive to the duty cycle of ISRJ. Therefore, based on the comprehensive simulation results under different duty cycle conditions, the performance of BCS is still inferior to that of the CVEDNet proposed in this paper. These experimental results indicate that the proposed network can maintain consistent performance under varying jamming strengths while effectively achieving high $P_{d}$ and low $P_{fa}$. By training on a diverse dataset that includes various types of ISRJ, CVEDNet is able to learn the key feature differences between echo signals and ISRJ with different forwarding parameters and power levels. This ensures the accuracy and stability of signal separation by the network under varying JSR conditions.

**Figure 16:**
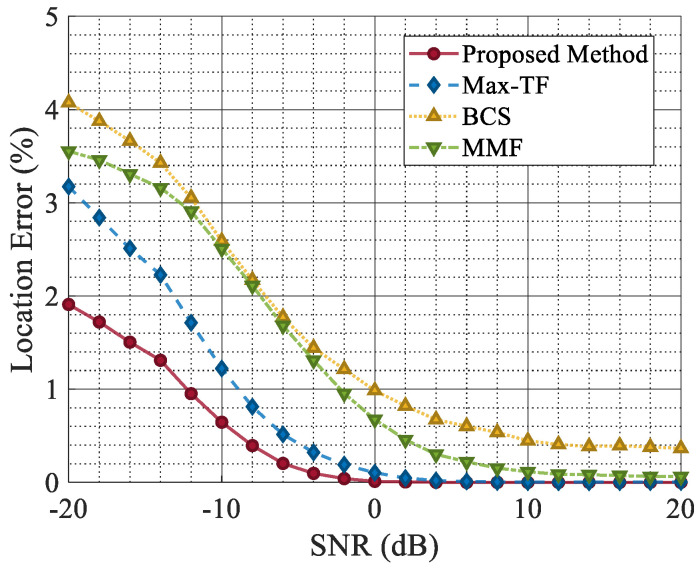
Location error with varying SNRs.

As shown in Figure 16, the localization error of all methods increased as the SNR decreased. Notably, the BCS exhibits a higher localization error compared to other methods. This is because the BCS relies on target recovery from jamming-free segments, rendering it more susceptible to misidentifying high-energy non-target signals as the target when the sidelobes of the received signal are prominent. Consequently, this misclassification leads to a deviation in the localization accuracy.

**Figure 17:**
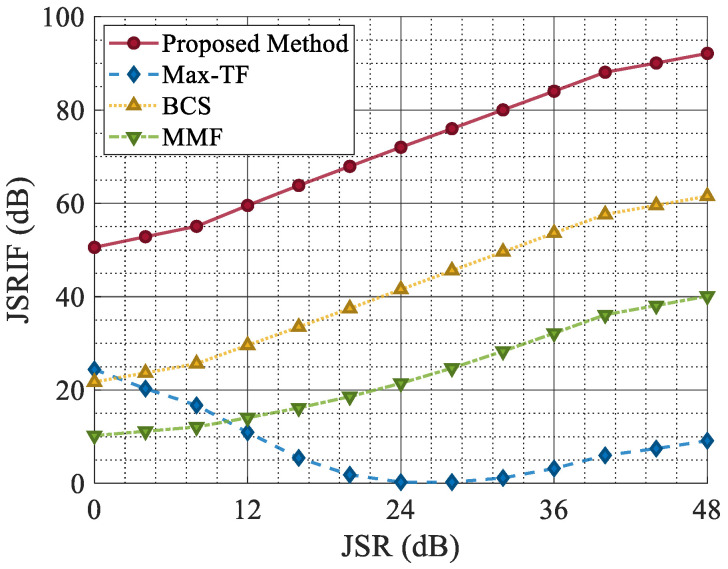
The JSRIF with varying JSRs.

Furthermore, as shown in Figure 17, except for the Max-TF, the JSRIF of other methods increased as the JSR increased. This is because the Max-TF leaves small residual jamming when filtering out the jamming signal. As the jamming intensity increases, the amplitude of the residual jamming also grows, affecting the CFAR results in the intermediate steps of Max-TF and ultimately rendering its JSRIF enhancement step ineffective. For the MMF, although the mismatched filter reduces the coherence between the receiver and the jamming, ISRJ retains partial coherence with the target signal. Consequently, the target amplitude after mismatched filtering inevitably decreases.

The experimental results above demonstrate that the proposed network consistently maintains a low localization error across the entire SNR range and exhibits superior jamming suppression performance in terms of the JSRIF over the entire JSR range.

### 4.8. Generalization Verification

Considering that the samples of the training data set all follow the ISRJ pattern of Figure 1, only instances where the ISRJ false target lags behind the real target are included. In this section, frequency modulation is used to generate more ISRJ patterns to verify the generalization performance of the CVEDNet proposed in this paper. Frequency-modulated ISRJ can generate false targets that are ahead of the real target. In the newly generated data, considering the role of the front-end filter of the radar receiver, the frequency-modulated ISRJ band is limited within the bandwidth of the radar signal. The total count of newly generated data featuring frequency-modulated ISRJ is 30240, while other metrics align with the parameters outlined in Table 1.

It should be noted that the network is not retrained in this section, that is, the data of the new ISRJ patterns does not participate in the network training process. The experiments in this section directly use the training results of CVEDNet in Section 4.3 to test the new dataset.

**Figure 18:**
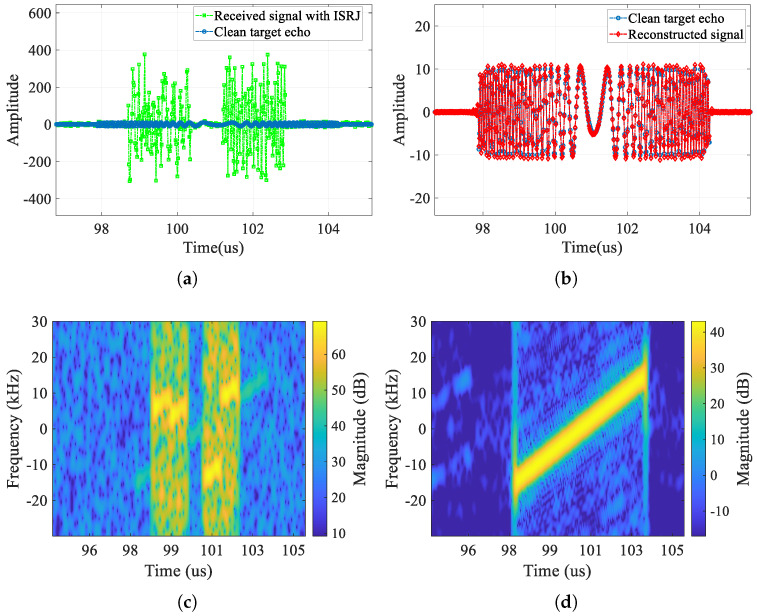
Waveform with frequency-modulated ISRJ when SNR is 0 dB and the JSR is 8 dB. (**a**) Received signal with frequency-modulated ISRJ and clean target echo, (**b**) clean target echo and the reconstructed signal, (**c**) time–frequency map of the received signal with frequency-modulated ISRJ, and (**d**) time–frequency map of the reconstructed signal.

**Figure 19:**
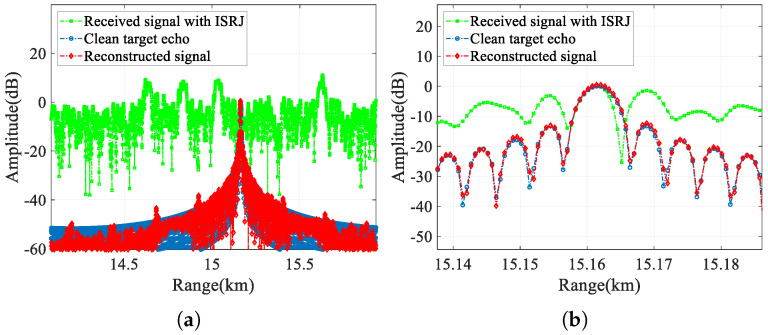
Pulse compression results with frequency-modulated ISRJ when SNR is 0 dB and the JSR is 8 dB. (**a**) Comparison in the range profile and (**b**) comparison in the enlarged range profile.

First, we compare the time domain (real part), time–frequency domain, and range-pulse-compression results of the jammed signal with frequency-modulated ISRJ, the clean target echo, and the reconstructed signal output by CVEDNet in Figure 18 and Figure 19. From the time–frequency domain and pulse-compression results, it can be seen that frequency-modulated ISRJ can produce false targets that are ahead of the real targets. Nevertheless, CVEDNet trained with the traditional ISRJ dataset can still achieve the jamming countermeasure and target echo reconstruction under frequency-modulated ISRJ and maintains good performance.

**Figure 20:**
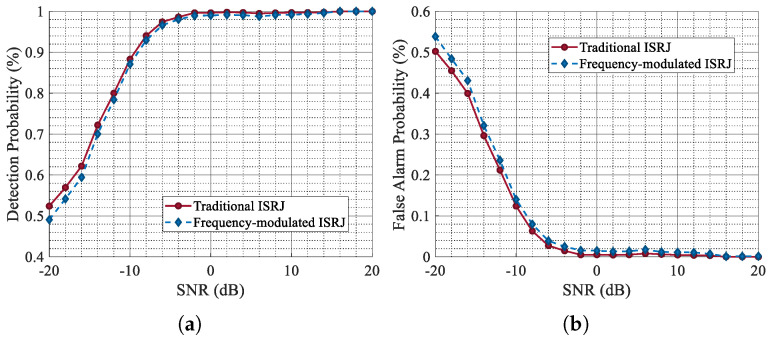
Comparison of $P_{d}$ and $P_{fa}$ for different dataset. (**a**) $P_{d}$ for the comparison. (**b**) $P_{fa}$ for the comparison.

Secondly, quantitative indicators are used to evaluate the performance of the proposed network on the new dataset from the perspective of target detection probability and waveform quality. Figure 20 demonstrates the curves of $P_{d}$ and $P_{fa}$ of the new dataset as a function of SNR. Table 5 shows the time-domain evaluation indicators of the waveform reconstruction quality of the new dataset.

For the frequency-modulated ISRJ dataset, the target detection performance of the echo data processed by CVEDNet remains largely consistent with that of the original dataset, with only a slight decrease. The experimental results show that CVEDNet can accurately capture ISRJ features and shows good generalization capabilities.

The evaluation metrics SISNR and SISDR-IF of the reconstructed waveform output by CVEDNet decreased by 2.25 dB and 3.32 dB, respectively. This result shows that for frequency-modulated ISRJ data that did not participate in the training process, CVEDNet can effectively attenuate ISRJ and noise to achieve echo waveform reconstruction, but the quality of waveform reconstruction will decrease.

### 4.9. Discussion

This work introduces an end-to-end ISRJ suppression network tailored for low-SNR applications. Rooted in the concept of signal separation and joint feature extraction, the proposed CVEDNet achieves radar echo waveform reconstruction while mitigating ISRJ and noise. Experimental results underscore the network’s superiority over conventional jamming countermeasure methods, as evidenced by network training outcomes, waveform reconstruction quality, and radar detection performance across varying SNRs and JSRs. Furthermore, the generalization ability of CVEDNet is validated across different ISRJ patterns.

Undoubtedly, the jamming countermeasure of radar poses a multifaceted challenge. This study simplifies the radar’s operational mode to showcase the proposed network’s effectiveness in ISRJ countermeasure under specific conditions. In practical radar operations, both the operational mode and waveform are subject to change to enhance the countermeasure efficacy. Additionally, radar systems often encounter not just singular but composite jamming, alongside challenges such as clutter background, nonlinearity, and other adverse factors that impact countermeasure effectiveness. Notably, this study focuses only on scenarios featuring a single radar echo. When multiple radar echoes are present, the increased complexity poses a heightened challenge for jamming countermeasures.

Hence, future endeavors should aim to enhance the network’s adaptability to diverse radar operational modes and broaden its scope to address more jamming types and more complex scenarios.

## 5. Conclusions

Starting from the perspectives of mixed signal decomposition and signal reconstruction, an end-to-end ISRJ countermeasure network was proposed in this study that effectively addresses the challenge of ISRJ suppression under low-SNR conditions. Our method employs a complex-valued encoder–decoder structure, allowing for the direct processing of complex-valued time-domain signals and noise suppression. Building upon noise suppression, the stacked dual-branch structure achieves a gradual separation of echo and ISRJ based on high-dimensional features. Furthermore, a multi-domain combined loss function is designed to enhance the echo reconstruction performance. Jamming suppression experiments were conducted on signals carrying ISRJ with varying JSRs and jamming proportions under different SNRs. The simulation results illustrate that the proposed method outperforms other techniques in target detection, showcasing superior performance. This study focused on the performance evaluation in single-target scenarios. Future work will be dedicated to validating the proposed method in multi-target scenarios and enhancing the model’s performance and generalization capabilities for more jamming types.

## Figures and Tables

**Table 1:** Simulation parameters of the radar and jammer.

Parameters		Values
Radar transmitting signal (LFM)	Carrier frequency $f_{c}$	16 GHz
	Pulse width $T_{c}$	6.4 µs
	Bandwidth $B_{c}$	30 MHz
	Sampling frequency $F_{s}$	60 MHz
Target signal	Target amplitude $A_{\mathrm{target}}$	1∼10
	Target range $R_{\mathrm{target}}$	0∼30 km
	SNR	−20∼20 dB
ISRJ signal	JSR	0∼50 dB
	Slice width $W_{s}$	0.512∼1.536 µs
	Forwarding times	2∼5 times

**Table 2:** The parameters of the network architecture.

Layer	Output Size	Parameter
Input	768×1	-
Encoder	95×512	1.07 M
BottleNeck	95×128	0.13 M
DBCB group1	95×128	0.40M×8
DBCB group2	95×128	0.40M×8
DBCB group3	95×128	0.40M×8
Expansion	95×512	0.13 M
Decoder	768×1	0.01 M
Total	-	11.02 M

**Table 3:** Evaluation criteria of the reconstructed signal.

Signal	Symbol	Parameter	Value (dB)
Target Echo	$PSLR_{r}$	range PSLR	−13.43
	$ISLR_{r}$	range ISLR	−9.82
	$PSLR_{v}$	velocity PSLR	−13.27
	$ISLR_{v}$	velocity ISLR	−9.75
Reconstructed Signal	$PSLR_{r}$	range PSLR	−13.18
	$ISLR_{r}$	range ISLR	−10.07
	$PSLR_{v}$	velocity PSLR	−13.45
	$ISLR_{v}$	velocity ISLR	−7.42

**Table 4:** Comparison of time-domain evaluation metrics for different loss functions.

Loss Function	SISDR (dB)	SISNR (dB)	SISDR-IF (dB)
MSE	12.74	12.73	41.33
MSE+MAE	13.47	13.46	42.05
MSE+Smooth L1 [25]	13.25	13.25	41.84
Multi-Domain Combined Loss (Proposed)	**14.13**	**14.13**	**42.72**

**Table 5:** Time-domain evaluation metrics for frequency-modulated ISRJ.

Dataset	SISDR (dB)	SISNR (dB)	SISDR-IF (dB)
Frequency-modulated ISRJ	11.88	11.88	39.40

## Data Availability

The data presented in this study are available on request from the corresponding author.
